# Phytochemical constituents of *Camellia osmantha* fruit cores with antithrombotic activity

**DOI:** 10.1002/fsn3.2769

**Published:** 2022-03-21

**Authors:** Li Yang, Gui‐Liang Xie, Jin‐Lin Ma, Xiao‐Qiong Huang, Yao Gu, Lei Huang, Hai‐Yan Chen, Xi‐Lin Ouyang

**Affiliations:** ^1^ Guangxi Key Laboratory of Special Non‐wood Forest Cultivation and Utilization Guangxi Zhuang Autonomous Region Forestry Research Institute Nanning China; ^2^ Department of Pharmacy Gannan Healthcare Vocational College Ganzhou China; ^3^ 74654 College of Public Health and Management Youjiang Medical University for Nationalities Baise China

**Keywords:** anticoagulant, *Camellia osmantha*, flavonoids, polyphenols, saponins, UPLC‐QTOF/MS

## Abstract

*Camellia osmantha* is a new species of the genus *Camellia* and is an economically important ornamental plant. Its activity and ingredients are less studied than other *Camellia* plants. This study investigated the antithrombotic effect and chemical components of *C. osmantha* fruit cores using platelet aggregation assays and coagulation function tests. The cores of *C. osmantha* fruits were extracted with ethanol to obtain a crude extract. The extract was dissolved in water and further eluted with different concentrations of methanol on an MCI resin column to obtain three fractions. These samples were used for antithrombotic activity tests and phytochemical analysis. The results showed that the extract and its fractions of *C. osmantha* have strong antithrombotic activity, significantly reducing the platelet aggregation rate and prolonging the thrombin time (TT). The total saponins, flavonoids, and polyphenols in the active fractions may be responsible for the antithrombotic activity. The chemical constituents were analyzed by ultra‐performance liquid chromatography‐quadrupole time‐of‐flight mass spectrometry (UPLC‐QTOF/MS). Twenty‐three compounds were identified rapidly and accurately. Among them, ellagic acid, naringenin, and quercetin 3‐*O*‐glucuronide may be important antithrombotic constituents. Furthermore, interactions between these compounds and the P2Y1 receptor were investigated via molecular modeling, because the P2Y1 receptor is a key drug target of antiplatelet aggregative activity. The molecular docking results suggested that these compounds could combine tightly with the P2Y1R protein. Our results showed that *C. osmantha* fruit cores are rich in polyphenols, flavonoids, and saponins, which can be developed into a promising antithrombotic functional beverage for the prevention and treatment of cardiovascular and cerebrovascular diseases.

## Graphical abstract

The extract of *C. osmantha* has good antithrombotic activity. The total saponins, flavonoids, and polyphenols may be responsible for the antithrombotic activity. Ellagic acid, naringenin, and quercetin 3‐*O*‐glucuronide combined tightly with the P2Y1R protein may be important antithrombotic constituents. *C. osmantha* fruit cores can be used as a promising antithrombotic functional beverage.

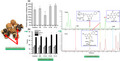

## 1 INTRODUCTION

Coagulation factors and platelets play important roles in maintaining the dynamic equilibrium in the human body (Dahlback, 2005). Platelet dysfunction, such as increased aggregation, and coagulation can lead to embolic heart and brain diseases including coronary heart disease, angina pectoris, and myocardial infarction (Wendelboe & Raskob, 2016). Some *Camellia* plant extracts have the potential to prevent a variety of cardiovascular and cerebrovascular diseases caused by hardening of blood vessels (Bansal et al., 2012; Chou et al., 2018), due to their rich content of bioactive compounds including triterpenoids and their glycosides, flavonoids, fatty acids, steroids, and lignins, which are obtained from the core, shell, flowers, roots, or leaves (Li et al., 2014; Lu et al., 2000; Zong et al., 2015). These components are implicated in the antitumor, antioxidation, hypolipidemic, glucosidase‐inhibiting, antifertility, antibacterial, and anti‐inflammatory activities of these plants (Imran et al., 2019; Jin, 2012; Ye et al., 2014; Zhou et al., 2014).

*Camellia osmantha* is an economically important ornamental plant of the genus *Camellia* with a high oil yield, a new species native to Guangxi, China (Ma et al., 2012). Its fruit cores are used as an important non‐*Camellia* tea in the minority region of Guangxi folklore and are consumed as a beverage because of their specific efficacy in preventing cardiovascular disease. It has been reported that polyphenols, saponins, and catechins in *Camellia* plants have the potential to prevent cardiovascular and cerebrovascular diseases (Bansal et al., 2012). To date, no definitive evidence has been found to confirm that extracts of *C. osmantha* can inhibit the activation of coagulation factors and platelets, and prevent the conversion of fibrinogen into fibrin. Thus, the antithrombotic activity and the possible chemical compositions of *C. osmantha* fruit cores were determined by an activity‐directed approach in the present study. Prothrombin time (PT), activated partial thromboplastin time (APTT), thrombin time (TT), and platelet aggregation rate were determined to explore the anticoagulant effect of *C. osmantha* fruit cores. Possible antithrombotic chemical constituents were inferred by UPLC‐QTOF/MS, and docking results were further verified by developing a pharmacophore model that clarified the key features required for an optimal P2Y1R affinity.

## 2 MATERIALS AND METHODS

### 2.1 Chemicals

The absorbance for determining the content of total flavonoids, total polyphenols, total polysaccharides, and total saponins was measured on a Shimadzu UV‐Vis spectrophotometer (UV‐1780, Shimadzu, Japan). Chemical composition analysis was performed on an Agilent 6545B Q‐TOF LC/MS using a Supelcosil ABZ+PLUS column (150 mm × 4.6 mm, 3 μm). Platelet aggregation induced by adenosine diphosphate (ADP) was measured using a semiautomatic platelet aggregometer (LBY‐NJ4, Beijing Precil Group, China). PT, APTT, and TT were tested by a C‐20004 semi‐auto coagulation analyzer (Beijing Precil Group, China). Coagulation test reagents were purchased from Shanghai TaiYang Biotechnology, China. DNS (3,5‐Dinitrosalicylic acid) and Folin–Ciocalteu's phenol reagent were purchased from Beijing Solarbio Science & Technology Co. Ltd. ADP was obtained from Ark Pharm, Inc. (Libertyville, IL, USA). Rutin, glucose, gallic acid, and saponin were purchased from Energy Chemical Technology Co., Ltd (Shanghai, China). Vanillin, perchloric acid, acetic acid, aluminum nitrate, sodium hydroxide, and other reagents were of analytical grade.

### 2.2 Plant material

*Camellia osmantha* fruit cores were collected during maturity (September 2019) in Nanning, Guangxi, and identified by Professor Jin‐Lin Ma of the Guangxi Zhuang Autonomous Region Forestry Research Institute, one of the discoverers of *C. osmantha*. The samples were smashed and passed through an 80‐mesh sieve after being dried in an oven at 60°C. The cores of *C. osmantha* fruit (200 g) were extracted three times with 80% analytical grade ethanol for 1 h with normal ultrasound‐assisted extraction (300 w, 40 Hz) and filtered. The filtrates were combined and dried under reduced pressure below 60°C to give a brown solid crude extract (CE, 2.15 g). The CE (1.8 g) was dissolved in water and was introduced into an MCI‐gel CHP20P, and then eluted with different concentrations (0%, 50%, and 100%) of methanol to obtain fraction A (Fr‐A, 0.6 g), fraction B (Fr‐B, 0.48 g), and fraction C (Fr‐C, 0.35 g). All samples including CE and three fractions were dried and stored at 4°C before performing the platelet aggregation assay, coagulation function test, and phytochemical analysis.

### 2.3 Animals

Wistar rats, weighing 220–250 g, were obtained from the Animal Experiment Center of Youjiang Medical University for Nationalities, Baise, China. The animals were placed in an environment with a relative humidity of 40%–70% at a temperature of 25°C, and fed freely for 2 days with food and water before use.

### 2.4 Platelet aggregation assay

Platelet aggregation assays were performed according to the methods reported in the literature (Gao et al., 2014). Wistar rats were anesthetized with 1% pentobarbital. Blood samples collected from the abdominal aorta were placed in a centrifuge tube (3.8%, w/v) with sodium citrate at a ratio of blood:anticoagulant = 9:1. The supernatant (platelet‐enriched plasma, PRP) was obtained by centrifugation at 160 *g* for 10 min at 25°C. The remaining blood sample was continuously centrifuged at 2000 *g* for 10 min to obtain platelet‐poor plasma (PPP). The platelet aggregation assay was performed by adding different extracts (1 mg/ml) to clean test cups and by inducing ADP at a final concentration of 5 μM. The maximum aggregation rate (MAR) within 6 min was observed by using physiological saline (9 mg/ml) as the negative group and aspirin (66 μg/ml) as the positive control. The aggregation inhibition rate (AIR) was calculated as follows:

(1) $$\text{AIR}\left(\%\right)=\frac{\left(\text{NCAR}‐\text{EAR}\right)}{\text{NCAR}}\times 100\%$$

EAR, Experimental group aggregation rate; NCAR, No‐treatment control group aggregation rate.

### 2.5 Coagulation function test

To determine the coagulation function of *C. osmantha* fruit cores in rats, PT, APTT, and TT assays were used to evaluate the coagulation effect. Fresh blood mixed with an anticoagulant in a 9:1 ratio collection from the rat aorta was centrifuged at 633 *g* for 15 min to obtain plasma. The CE (0.2 μl, 1 mg/ml) was added to 0.4 μl of plasma to obtain mixed plasma. The assay was performed according to the kit requirements. Briefly, 0.6 μl of plasma was incubated at 37°C for 3 min, and then a prewarmed PT reagent (0.6 μl) at 37°C was added to record the PT. Plasma (0.6 μl) was added to 0.6 μl of prewarmed APTT reagent at 37°C and incubated for 5 min at 37°C, and then prewarmed 0.6 μl of CaCl_2_ (0.025 mol/L) was added. The clotting time was recorded for TT after the prewarmed plasma was mixed with the TT reagent at 37°C. Three fractions were measured according to the above method. Each sample was measured three times.

### 2.6 Determination of total flavonoid, polyphenol, polysaccharide, and saponin contents

#### 2.6.1 Total flavonoids

The total flavonoid content (TFC) was determined using a method described in the literature (Li et al., 2012). Four samples (10 mg/ml), including CE or Frs‐A to C, were placed in a 10‐ml tube and then 0.15 ml of 5% NaNO_2_ was added. After 6 min, 0.15 ml of 10% Al(NO_3_)_3_ was added. Then, 2 ml of 4% NaOH was added another 6 min later, and the total volume adjusted to 5 ml with distilled water. The absorbance of the mixture was measured through a UV‐1780 spectrophotometer at 510 nm. Each test was repeated three times and the results were averaged.

#### 2.6.2 Total polyphenols

The total polyphenol content (TPPC) of the *C. osmantha* fruit core extracts was measured by a colorimetric assay primarily based on procedures described by Ordóñez‐Santos with a few modifications (Ordonez‐Santos et al., 2017). Briefly, 0.5 ml of CE or Frs‐A to C (10 mg/ml) was mixed with 1 ml of Folin–Ciocalteu's phenol reagent. After 3 min, 1 ml of saturated sodium carbonate solution was added to the mixture and adjusted to 10 ml with distilled water. The reaction was kept in the dark for 60 min before the absorbance was read with a UV‐1780 spectrophotometer at 760 nm. Gallic acid was used to construct the standard curve (0.01–0.4 mmol/l).

#### 2.6.3 Total polysaccharides

Total polysaccharide content (TPSC) was measured by a sulfuric acid–phenol spectrophotometric assay. A total of 2.0 ml of CE or Frs‐A to C (10 mg/ml) was placed into a stoppered tube, followed by a 5% phenol solution (1.0 ml). Then, 5.0 ml of concentrated sulfuric acid was added immediately, incubated for 30 min in a 40°C water bath, removed from the tube, and placed in a cold‐water bath for 5 min. The absorbance of each solution was measured at a wavelength of 490 nm. The reference solution was prepared with 2.0 ml of double‐distilled water. A standard curve was drawn with the concentration of the glucose diluent (4–20 μg/ml) plotted on the abscissa and the absorbance was plotted on the ordinate.

#### 2.6.4 Total saponin

The total saponin content (TSC) was measured by a vanillin–perchloric acid spectrophotometric assay (Xiao et al., 2014). The solution of CE or Frs‐A to C (10 mg/ml) was prepared in methanol (0.2 ml of aliquot) and was added to the colorimetric tube. After methanol solvent removal at 80°C, 0.5 ml of vanillin–acetic acid solution (5 mg/ml) and 1.5 ml of perchloric acid were added. The reaction mixture was incubated at 70°C for 15 min and then cooled and diluted with acetic acid to 10 ml. After 10 min, the absorbance of the diluted solution was measured at 540 nm with a UV‐1780 spectrophotometer, which was normalized against a solution of the reagents without the sample. The standard curve based on saponin (11.43–68.58 μg/ml) was quantified. All samples were tested three times.

### 2.7 UPLC‐ESI‐QTOF‐MS analysis

Chemical compositions of the *C. osmantha* fruit cores were determined on an Agilent 6545B Q‐TOF LC/MS according to our previous experimental procedures (Ouyang et al., 2020).

### 2.8 Molecular docking

The structure buildings of ellagic acid, naringenin, and quercetin 3‐*O*‐glucuronide were obtained from the PubChem Database (https://pubchem.ncbi.nlm.nih.gov/). These molecular ligands were generated by AutodockTools1.5.6 to add hydrogen and charge and detect the root of the ligand. The optimized structures were used for all subsequent calculations. The X‐ray crystal structure of the P2Y1R protein in complex with the receptor antagonist MRS2500 (PDB ID: 4XNW) was obtained from the Research Collaboratory for Structural Bioinformatics (RCSB) Protein Data Bank (www.rcsb.org). Autodock vina was used for semiflexible docking, and the best affinity conformation was selected as the final docking conformation. The active site was defined as including all atoms within a 6.5 Å radius of the cocrystallized ligand, and the default parameters were used.

## 3 RESULTS AND DISCUSSION

### 3.1 In vitro aggregation inhibition assay of CE and each fraction

**FIGURE 1:**
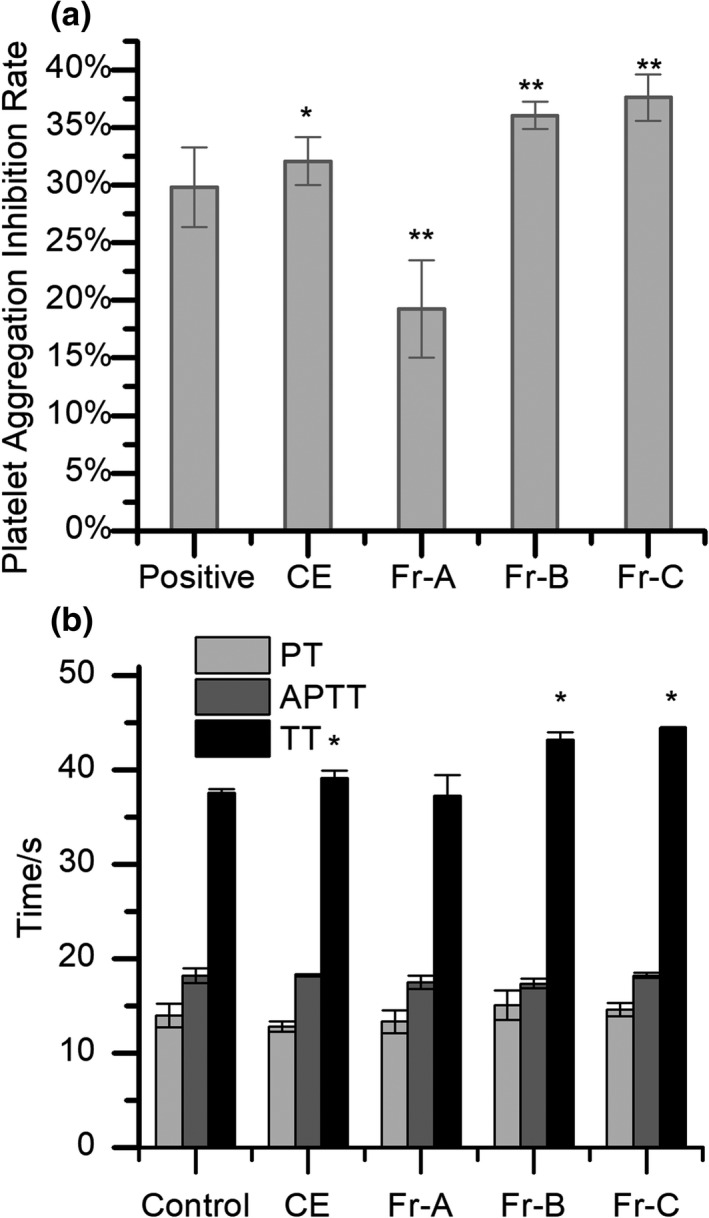
Effects of different fractions from *Camellia osmantha* fruit cores on coagulation index (a) and platelet aggregation (b)

Platelet aggregation function is the cardinal biological parameter used to evaluate antithrombotic activity. AIRs of CE and three fractions are shown in Figure 1a. The AIR of CE was 32.07%, higher than that of aspirin (*p* = .064), a well‐known platelet aggregation inhibitor with an AIR at 29.80%. Among the fractions, Fr‐C exhibited the best aggregation inhibition effect with an AIR of 37.61%, followed by Fr‐B with an AIR of 36.04%. Moreover, Fr‐B and Fr‐C had significantly higher AIRs than CE and fraction A. Thus, Fr‐B and Fr‐C have significant inhibitory effects on platelets and may be active fractions for antithrombotic applications.

### 3.2 Coagulation effects of CE and each fraction

Routine coagulation assays are important ways to evaluate the antithrombotic activities of compounds. APTT and PT reflect the status of the endogenous coagulation and exogenous coagulation systems, respectively (Jia et al., 2018). TT reflects the ability to convert fibrinogen to fibrin. As shown in Figure 1b, no significant differences were found between the extracts and the control group for PT and APTT, indicating that the anticoagulation effect may not be related to either the endogenous or exogenous coagulation pathways. Compared with the control group, CE, Fr‐B, and Fr‐C significantly prolong TT (*p* < .05), indicating that Fr‐B and Fr‐C may contain abundant active ingredients that could inhibit the conversion of fibrinogen to fibrin in preventing blood clotting. Moreover, the contents of anticoagulants in Fr‐B and Fr‐C were significantly enriched after extraction compared with CE. Thus, Fr‐B and Fr‐C of *C. osmantha* could prevent blood coagulation by affecting the fibrinolytic system.

### 3.3 Determination of total saponins, flavonoids, polyphenols, and polysaccharides

**FIGURE 2:**
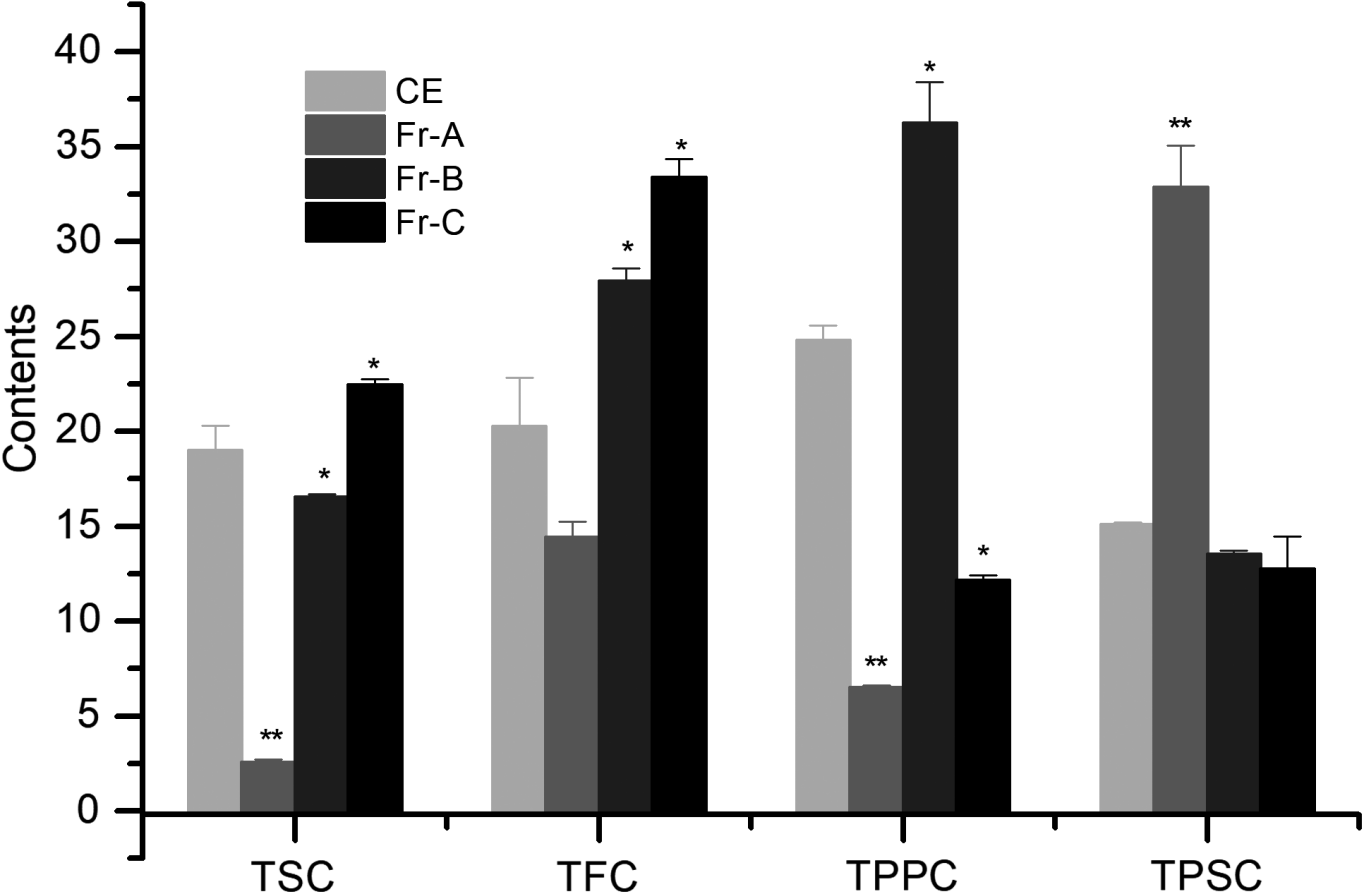
Comparison of total saponins content (TSC), total flavonoids content (TFC), total polyphenols content (TPPC), and total polysaccharides content (TPSC)

Different plants parts of genus *Camellia* have confirmed the beneficial effects for cardiovascular health for their corresponding chemical constituents, for example, *Camellia* oil is beneficial to cardiovascular health because of the presence of unsaturated fatty acids. Green tea can reduce atherosclerosis and lipid peroxidation mainly due to catechins, epigallocatechin gallate (EGCG), and other polyphenols (Basu & Lucas, 2007). To explore the composition of Fr‐B and Fr‐C, total saponins, flavonoids, polyphenols, and polysaccharides were determined by UV‐Vis spectrophotometric methods. As shown in Figure 2, Fr‐A showed a higher polysaccharide content than CE and other fractions, while Fr‐A had the weakest antiplatelet aggregation ability found in Figure 1a, suggesting that the antithrombotic activity of *C. osmantha* fruit cores may not be caused by polysaccharides. The contents of flavonoids and polyphenols in Fr‐B were much higher than those in the other fractions, while its content of total polysaccharides was lower than that in Fr‐A. Especially, the content of total polyphenols in Fr‐B was 36.23 ± 2.18% gallic acid equivalents (GAE), significantly higher than that reported by Anesini et al. (Anesini et al., 2008). Similarly, the contents of polyphenols and flavonoids in Fr‐C were higher than those in CE and Fr‐A. Fr‐B and Fr‐C showed considerable antiplatelet aggregation ability, but their chemical compositions were not consistent. These results suggested that polyphenols and flavonoids in Fr‐B, as well as saponins and flavonoids in Fr‐C, may be the main contributors to the anticoagulant activity of the *C. osmantha* fruit cores.

Moreover, Spearman correlation analysis performed for the content of each fraction and AIR indicated a statistically significant correlation between total saponins and AIR (Table 1). A moderate correlation between the contents of flavonoids or polyphenols and AIR was found, whereas no such correlation was found for flavonoids and polyphenols. It has also been observed that the polysaccharide content and AIR are negatively correlated. Regarding saponins in Fr‐C and polyphenols in Fr‐B, a marked increase in their concentration and a positive correlation with AIR suggest that saponins and polyphenols might represent different active ingredients in *C. osmantha* fruit cores. Hence, it is easy to draw a preliminary conclusion, which is that *C. osmantha* fruit cores have antithrombotic effects, which may be mediated by affecting the aggregation of platelets; among the various fractions, Fr‐B and Fr‐C have good active effects. The determination of total flavonoids, total phenols, total saponins, and total sugars revealed that Fr‐B is mainly comprised of polyphenols and flavonoids, while Fr‐C is a mixture of polyphenols and flavonoids.

**TABLE 1:** Pearson's correlation coefficients for total saponin contents (TSC), flavonoids (total flavonoid content (TFC)), polyphenols (total polyphenol content (TPPC)), and polysaccharides (total polysaccharide content (TPSC)) of *Camellia osmantha* fruit cores and aggregation inhibition rate (AIR)

	AIR	TSC	TFC	TPPC	TPSC
AIR	1	.912*	.794	.797	−.983**
TSC		1	.475	.475	−.887
TFC			1	.576	−.826
TPPC				1	−.871
TPSC					1

*p < .05, **p < .01.

### 3.4 HPLC‐ESI‐QTOF‐MS analysis

The specific chemical compounds of Fr‐B and Fr‐C were further analyzed by UPLC‐QTOF‐MS. According to the retention time, the precursor *m/z* of the positive and negative ions, the isotope ratio, and the reference compounds confirmed from the *Camellia* genus, 23 compounds were initially identified that contained polyphenols, flavonoids, triterpenes, organic acids, etc. (Table 2). Although MCI‐gel was used as the separation material for isolation, 18 compounds were still found in both Fr‐B and Fr‐C. UV‐Vis analysis of most peaks confirmed that these compounds had maximum absorption only at 278 nm with a distinct shoulder at 283 nm, but had no absorption in the visible region, suggesting they might be polyphenols (Uchida et al., 2016). A small part of the peaks also has a maximum absorption at 330–340 nm, which suggests that they may be flavonoids. These findings are consistent with a previous analysis on the content of total polyphenols in Fr‐B and Fr‐C.

**TABLE 2:** Analytical result of the chemical constituents of fraction B (Fr‐B) and fraction C (Fr‐C) by ultra‐performance liquid chromatography‐quadrupole time‐of‐flight mass spectrometry (UPLC‐QTOF/MS)

No.	Source (Fr.)	*t*_R_/min	Negative ion mode		Positive ion mode		Molecular formula	Proposed compound	Classification	References
			Adducts	*m/z*/Diff (ppm)	Adducts	*m/z*/Diff (ppm)				
1	B	0.823	[M−H]^−^	483.0757 (2.38)	[M+NH_4_]^+^	502.1188 (0.31)	C_20_H_20_O_14_	Digalloyl‐glucose	Polyphenol	Liu et al. (2012); Ryu et al. (2017)
2	B	1.073	[M−H]^−^	635.0857 (3.24)	[M+NH_4_]^+^	654.12939(0.68)	C_27_H_24_O_18_	Trigalloyl‐glucose	Polyphenol	Ryu et al. (2017); Wei et al. (2019)
3	B	1.470	[M−H]^−^	289.0707 (1.01)	[M + H]^+^	291.0861 (0.21)	C_15_H_14_O_6_	Catechin or epicatechin	Polyphenol	Wang et al. (2017)
4	B	1.472	[M−H]^−^	521.2006 (2.26)	[M+Na]^+^	545.1990 (0.37)	C_26_H_34_O_11_	Icariside E5	Lignan	Zeng et al. (2020)
5	B &C	2.221	[M−H]^−^	787.0958 (4.15)	[M+Na]^+^	806.1392 (1.83)	C_34_H_28_O_22_	Myricetin 3‐*O*‐glucosylrutinoside	Flavonoid	Wu et al. (2016)
6	B &C	2.338	[M−H]^−^	197.0444 (1.13)	[M + H]^+^	199.0599 (0.21)	C_9_H_10_O_5_	Syringic acid	Organic acid	Hong et al. (2019)
7	B &C	3.203	[M−H]^−^	297.0969 (1.04)	[M+Na]^+^	321.0935 (0.94)	C_14_H_18_O_7_	Chakanoside I	Organic acid ester	Yoshikawa et al. (2008)
8	B &C	3.404	[M−H]^−^	579.2389 (4.58)	/	/	C_27_H_32_O_14_	Naringenin	Flavonoid	Hou et al. (2013); Xiong et al. (2018)
9	B &C	3.553	[M−H]^−^	391.1385 (1.37)	[M+Na]^+^	415.1361 (0.21)	C_20_H_24_O_8_	5‐Dihydroxydihydrostilbene 4’‐*O*‐β‐D‐glucopyranoside	Terpene lactone	Cuc et al. (2020)
10	B &C	3.969	[M−H]^−^	755.2006 (1.89)	[M + H]^+^	757.2199 (1.22)	C_33_H_40_O_20_	Kaempferol‐3‐*O*‐galactose‐rutinoside	Flavonoid	Ryu et al. (2017)
11	B & C	4.636	[M−H]^−^	477.0641 (3.35)	[M+Na]^+^	501.0640 (0.04)	C_21_H_18_O_13_	Quercetin 3‐*O*‐glucuronide	Flavonoid	Zhou and Yang (2000)
12	B &C	4.901	[M−H]^−^	253.0683 (0.84)	/	/	C_15_H_10_O_4_	Chrysophanol	Anthraquinone	Ye et al. (2020)
13	B &C	5.001	[M−H]^−^	567.1687 (0.86)	[M+Na]^+^	591.1675 (0.92)	C_26_H_32_O_14_	Phloretin‐2‐*O*‐(‐D‐xylopyranosyl‐(1→6)‐D‐glucopyranoside)	Polyphenol, Dihydrochalcones	Ye et al. (2020)
14	B &C	5.051	[M−H]^−^	463.0850 (3.21)	[M + H]^+^	465.1054 (2.54)	C_21_H_20_O_12_	Myricitrin	Flavonoid	Ryu et al. (2017)
15	B & C	5.273	[M−H]^−^	300.9981 (1.99)	[M + H]^+^	303.0137 (0.09)	C_14_H_6_O_8_	Ellagic acid	Polyphenol	Ouyang et al. (2020)
16	B &C	5.284	[M−H]^−^	435.1275 (2.20)	[M+Na]^+^	459.1269 (0.73)	C_21_H_24_O_10_	Phloridzin	Polyphenol, Dihydrochalcones	Ye et al. (2020)
17	B &C	5.334	[M−H]^−^	623.2070 (1.53)	/	/	C_28_H_32_O_16_	Isorhamnetin‐3‐*O*‐neohesperidoside	Flavonoid	Ye et al. (2020)
18	B &C	5.517	[M−H]^−^	515.0404 (1.46)	/	/	C_25_H_24_O_12_	Isochlorogenic acid	Phenylpropanoid	Ye et al. (2020)
19	B &C	5.517	[M−H]^−^	593.1472 (3.72)	[M+Na]^+^	617.1480 (0.30)	C_27_H_30_O_15_	Kaempferol‐3‐*O*‐*p*‐coumaroyl‐glucose	Flavonoid	Ryu et al. (2017)
20	B &C	5.634	[M−H]^−^	461.1061 (2.78)	[M+Na]^+^	485.1058 (0.36)	C_22_H_22_O_11_	Kaempferide 3‐glucoside	Flavonoid	Wang et al. (2019)
21	B &C	6.072	[M−H]^−^	273.0749 (1.89)	/	/	C_15_H_14_O_5_	Phloretin	Flavonoid	Ye et al. (2020)
22	C	6.388	[M−H]^−^	315.0493 (1.67)	/	/	C_16_H_12_O_7_	Pollenitin	Flavonoid	Yang et al. (2018)
23	C	6.721	[M−H]^−^	285.0391 (1.35)	/	/	C_15_H_10_O_6_	3',4',5,7‐Tetrahydroxyflavone	Flavonoid	Wang et al. (2017)

**FIGURE 3:**
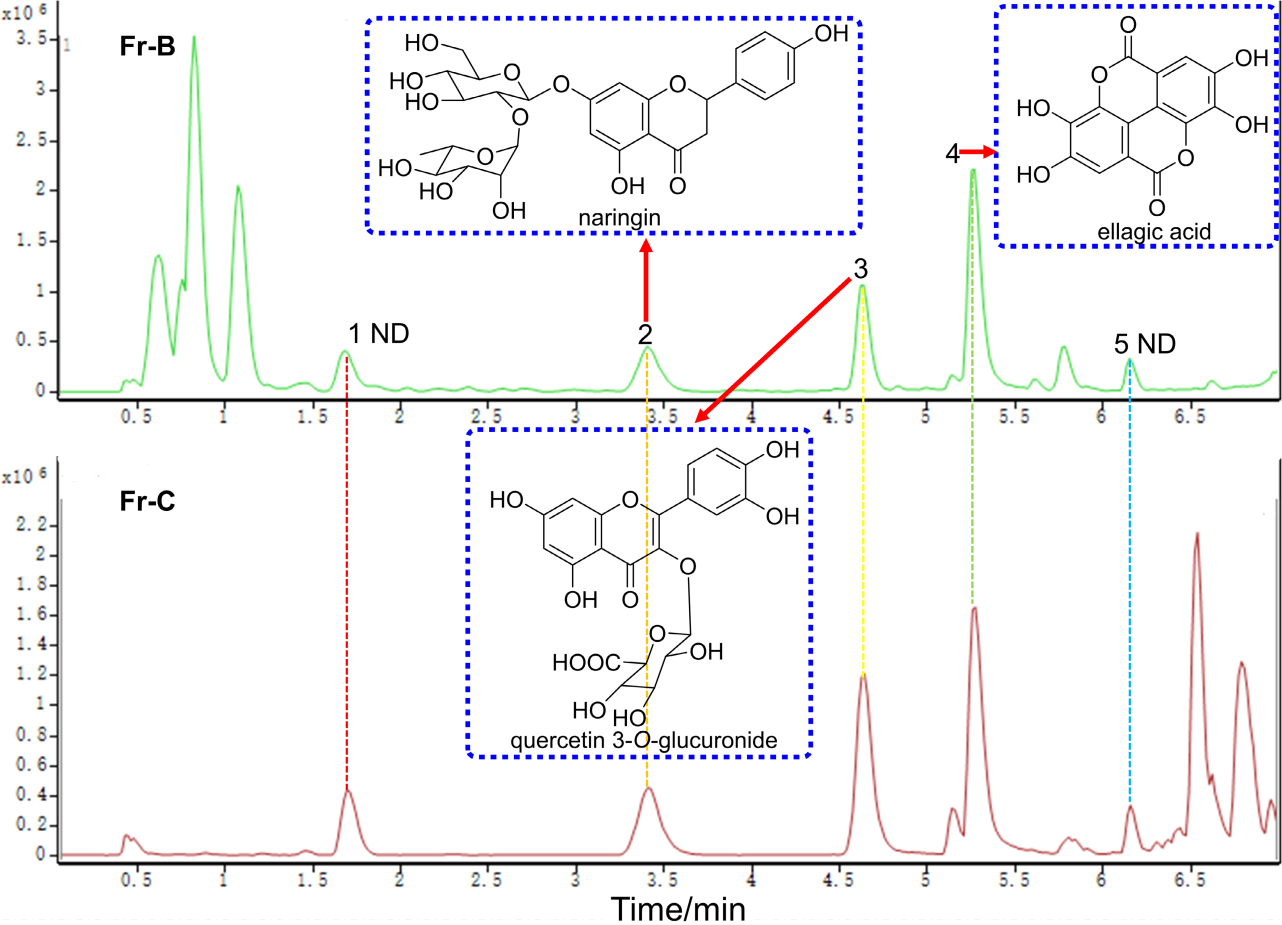
Comparison of base peak chromatogram of Fr‐B and Fr‐C (ND, not determined)

Figure 3 shows the base peak chromatograms (BPCs) of Fr‐B and Fr‐C in the negative ionization modes of analysis. The mass spectrum peaks of Fr‐B and Fr‐C have five major ion peaks in common (*t*_R_ = 1.694, 3.404, 4.636, 5.273, and 6.155 min), which correspond to naringenin (*t*_R_ = 3.404 min, a flavonoid), quercetin 3‐*O*‐glucuronide (*t*_R_ = 4.636 min, a flavonoid), and ellagic acid (*t*_R_ = 5.273 min, a polyphenol), respectively. Ellagic acid is a polyphenol component that can activate the inducers of coagulation factors XII and XI and it has anticoagulant effects. Ellagic acid can treat pathological arrhythmias, ventricular hypertrophy, and lipid peroxidation in rats with myocardial infarction caused by isoproterenol (Kannan & Quine, 2013). The peak at 5.273 min from Fr‐B showed a mass‐to‐charge ratio information with *m*/*z* at 300.9974 (calcd for C_14_H_6_O_8_ [M–H]^−^, 300.9984). In addition, peaks 1 and 5 showed two ultraviolet absorptions at 210 nm and 280 nm. They are preliminarily judged to be two phenolic compounds. However, their impossible structures could not be deduced because of the lack of more information. Moreover, although the extract of *C. osmantha* fruit cores had been proven to be rich in triterpenoids, few triterpenes have been identified due to the cleavage method of triterpenes previously described in the literature.

Thus, the active constituents of *C. osmantha* fruit cores may be polyphenols, flavonoids, and saponins rather than polysaccharides, which is consistent with the previous literature about the *Camellia* genus (Okinda Owuor et al., 2006).

### 3.5 Docking analysis

The P2Y1 receptor (P2Y1R) facilitates platelet aggregation and is thus an important potential antithrombotic drug target. The P2Y1R protein structure contains a binding site for the receptor antagonist MRS2500 in its seven‐transmembrane bundle, which also provides suitable pockets for numerous other ligands and nucleotide agonists of P2Y1R.

**FIGURE 4:**
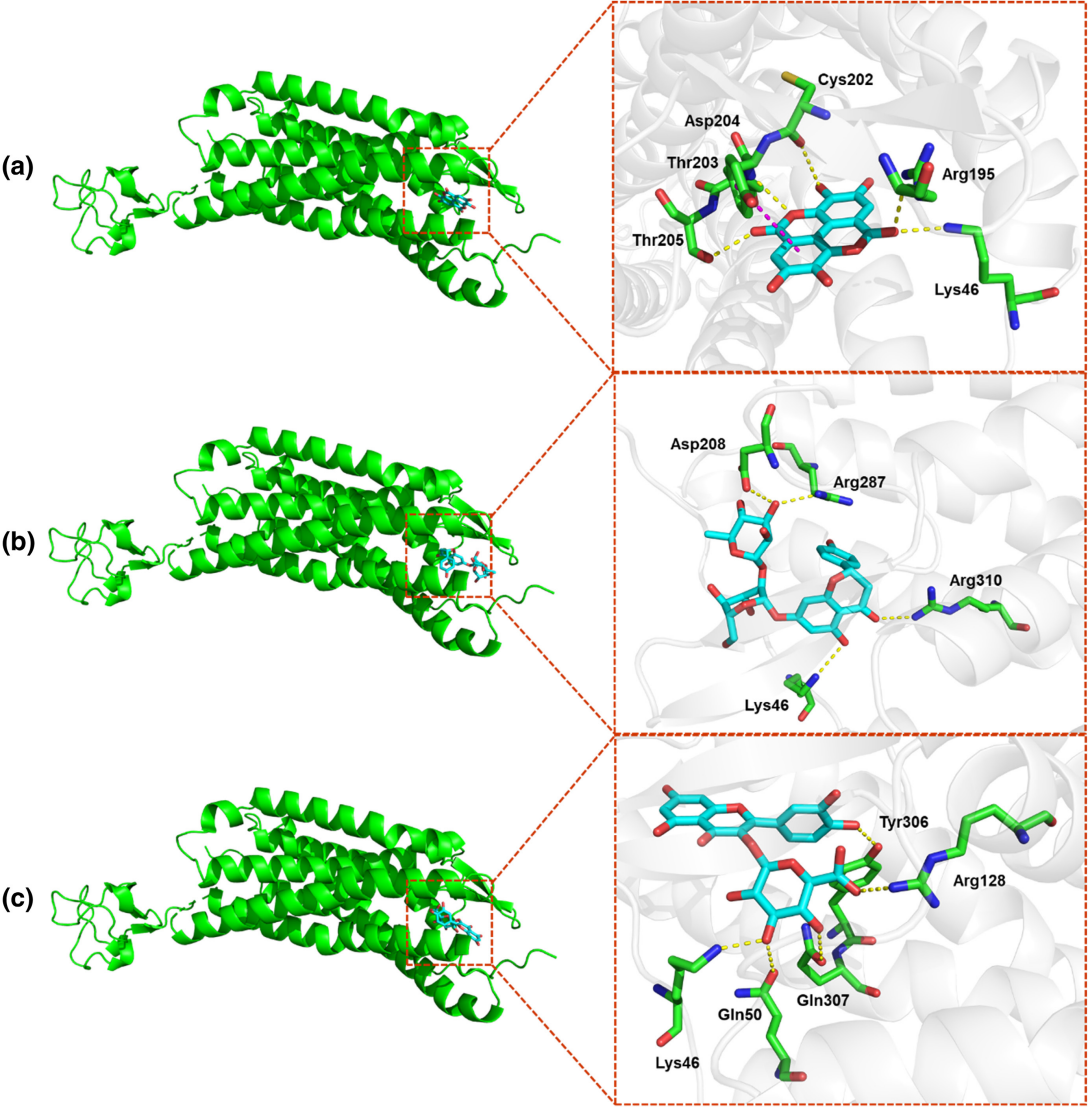
Amino acids of 4XNW interacting with ellagic acid (a), naringenin (b), and quercetin 3‐*O*‐glucuronide (c). Hydrogen bonds are shown as yellow dots and π–π bond is shown as pink dots

To further investigate the binding conformations of the active compounds with the 4XNW protein, molecular docking modeling was carried out using the LibDock method to dock ellagic acid, naringenin, and quercetin 3‐*O*‐glucuronide into the active sites of the 4XNW protein. The docked conformations of the best‐fit ligands were visualized: these fits extended deep into the active site pocket, forming several hydrogen bonds and hydrophobic interactions with the key residues of the active site (Figure 4). The docking results showed that hydrogen bond interactions played important roles in ligand–protein interactions. Ellagic acid could form five hydrogen bonds with five residues (Lys46, Arg195, Cys202, Asp204, and Thr205) and form a π–π bond with Thr203. Naringenin could form four hydrogen bonds with four residues (Lys46, Asp208, Arg287, and Arg310). Quercetin 3‐*O*‐glucuronide could form five hydrogen bonds with five residues (Lys46, Gln50, Arg128, Tyr306, and Gln307). Moreover, the binding energies of ellagic acid, naringenin, and quercetin 3‐*O*‐glucuronide to the 4XNW protein are −9.9, −8.6, and −9.9 kcal/mol, respectively, implying the formation of stable bond states. The above molecular docking results suggested that these compounds could tightly combine with the P2Y1R protein, consistent with the results of the P2Y1R protein inhibition assay. The specific molecular mechanism needs to be clarified in future studies.

## 4 CONCLUSIONS

Among the Guangxi folk, the cores of *C. osmantha* fruits have been widely considered to confer cardiovascular protection. However, the active components in the extracts of *C. osmantha* responsible for their antithrombotic effects are uncertain. According to the results obtained, both Fr‐B and Fr‐C could prolong TT and inhibit platelet aggregation compared with CE, which indicated that the contents of the active chemical components increased after the separation of MCI column. The ethanol extract of *C. osmantha* fruit cores displayed good antithrombotic activity mainly due to flavonoids, polyphenols, and saponins, which prevented blood clotting by affecting the fibrinolytic system. In addition, 23 compounds, including 6 polyphenols and 11 flavonoids, were identified by the HPLC‐QTOF‐MS equipped with high‐resolution mass detectors. The antithrombotic properties of *C. osmantha* fruit cores were associated with the presence of numerous flavonoids, polyphenols, and saponins. Ellagic acid, naringenin, and quercetin 3‐*O*‐glucuronide are the main compounds from the most activity fractions, which may have contributed to the most significant antithrombotic activity. Furthermore, molecular docking analysis revealed that, ellagic acid, naringenin, and quercetin 3‐*O*‐glucuronide showed stronger ability to interact with P2Y1 receptors. Thus, *C. osmantha* fruit cores possess the material basis for antithrombotic activity and can be developed into a promising antithrombotic functional beverage for the prevention and treatment of cardiovascular and cerebrovascular diseases and other thromboembolic diseases.

## CONFLICT OF INTEREST

The authors have declared no conflicts of interest for this article.

## AUTHOR CONTRIBUTIONS

**Li Yang:** Investigation (equal); Methodology (equal); Resources (equal). **Gui‐Liang Xie:** Formal analysis (equal); Methodology (equal); Writing – review & editing (equal). **Jin‐Lin Ma:** Resources (equal); Writing – review & editing (equal). **Xiao‐Qiong Huang:** Data curation (equal); Investigation (equal); Methodology (equal). **Yao Gu:** Data curation (equal); Investigation (equal). **Lei Huang:** Data curation (equal); Investigation (equal). **Hai‐Yan Chen:** Software (equal); Visualization (equal). **Xi‐Lin Ouyang:** Data curation (equal); Funding acquisition (equal); Project administration (equal); Writing – original draft (equal).

## ETHICAL APPROVAL

All animal‐related experiments were performed with the prior approval of the Experimental Animal Ethics Committee of Youjiang Medical University for Nationalities, Guangxi, P. R. China (Approval No. 02/2017).

## Data Availability

The datasets generated during and/or analyzed during the current study are available from the corresponding author upon reasonable request.
